# Responsive Dual-Targeting Exosome as a Drug Carrier for Combination Cancer Immunotherapy

**DOI:** 10.34133/2021/9862876

**Published:** 2021-08-31

**Authors:** Yuanyuan Fan, Yingshun Zhou, Meng Lu, Haibin Si, Lu Li, Bo Tang

**Affiliations:** College of Chemistry, Chemical Engineering and Materials Science, Collaborative Innovation Center of Functionalized Probes for Chemical Imaging, Key Laboratory of Molecular and Nano Probes, Ministry of Education, Shandong Normal University, Jinan 250014, China

## Abstract

Recently, combination immunotherapy, which incorporates the activation of the immune system and inhibition of immune escape, has been proved to be a new powerful strategy for more efficient tumor suppression compared to monotherapy. However, the major challenge is how to integrate multiple immune drugs together and efficiently convey these drugs to tumor sites. Although a variety of nanomaterials have been exploited as carriers for targeting tumor issues and the delivery of multiple drugs, their potential toxicity, immune rejection, and stability are still controversial for clinical application. Here, we proposed endogenic exosomes as drug carriers to deliver two antibodies acting as tumor-targeting molecules and block checkpoint inhibitors with specific response to the tumor microenvironment and costimulatory molecules for further improvement of therapeutic effect. The versatile exosomes exhibit excellent biocompatibility and provide a combination immunotherapy platform with synergistic advantages of activation of immune response and inhibition of immune escape.

## 1. Introduction

In recent years, cancer immunotherapy has attracted tremendous attention due to a number of successful clinical outcomes [1–3]. Initially, studies mainly focused on the strategies that activate and increase immune response, such as some drugs that promote the activation of antigen-presenting cells or amplification of naive T cells [4–6]. However, with the continuous in-depth studies, researchers found that tumor cells could escape from the attack of the immune system, leading to the failure of immune activation [7–10]. Therefore, a renewed interest has focused on inhibition of immune escape, which aims at rescuing the suppressed immune response by some block checkpoint molecules including programmed cell death protein 1 (PD-1), programmed cell death ligand 1 (PD-L1), and cytotoxic T lymphocyte-associated protein-4 (CTLA-4) [11, 12]. In addition, some researchers found that the combination of multiple immune drugs could greatly improve the effect of immunotherapy [13, 14]. However, it is difficult to obtain ideal therapeutic effects by direct injection of multiple drugs, because most of drugs have broad distribution which may lead to nonspecific drug release and reduction of synergistic effect [15, 16]. Thus, the development of versatile drug carriers is essential for loading multiple drugs used for combination therapy and, more importantly, for targeting modification to improved accumulation of drugs in tumor issues and reduce off-target effect.

A variety of nanomaterials has been designed as carriers for targeting tumor issues and delivery of multiple drugs [16–19]. By loading drugs on lumen or the surface of the nanomaterials, drugs can be enriched and assembled in the tumor site by active targeting (modification of some targeted molecules) or passive targeting (enhanced permeability and retention effect (EPR effect)), which is favorable for avoiding off-target effects. Moreover, drugs can escape from the clearance of the reticuloendothelial system by protection of nanomaterials and precise release in target sites [17, 20]. Although much progress has been made in delivering multiple immune drugs by nanomaterials, a number of challenges remain. For instance, potential toxicity and immune rejection of nanomaterials remain to be further studied. In addition, in the process of transport in blood, various proteins may be adsorbed on the nanomaterials, forming “protein corona,” to affect their stability and targeting ability [21, 22].

Recently, researchers discovered that exosomes, a kind of nanoscale membrane-bound vesicular particles, hold great potential in the delivery of clinical drugs [23–25]. Naturally, exosomes can provide inner space for drug payload, which show more flexibility in drug loading and effective delivery. Additionally, compared to other nanomaterials such as liposomes, polymers, and inorganic nanomaterials, exosomes originate from living cells and are with low immunogenicity and high biocompatibility [23]. More importantly, the issue for nanomaterials suffering from protein absorption is absent for exosomes [26]. All the above are beneficial for the stable transport of drugs in the blood and controlled drug release at the tumor site. Thus, we consider that as drug carrier, exosomes will provide new insights into tumor immunotherapy.

**Scheme 1:**
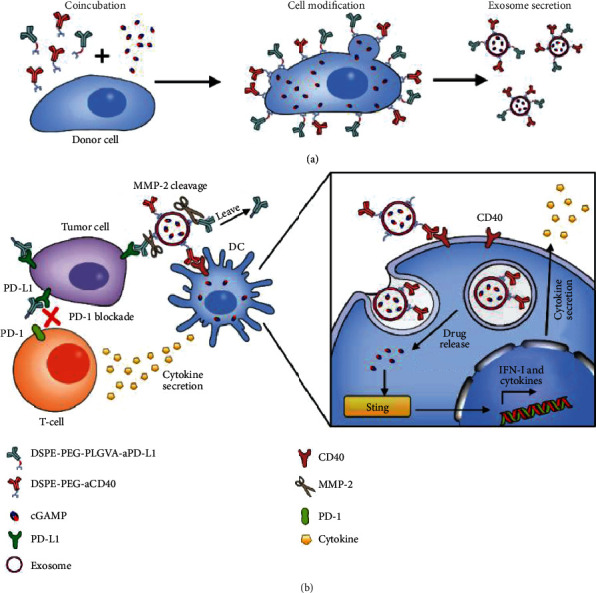
Schematic of dual-targeting and drug-loaded exosomes (cGAMP@dual-anti-Exos) for tumor immunotherapy. (a) The generation process of cGAMP@dual-anti-Exos. (b) The effect of activation of immune response and inhibition of immune escape by cGAMP@dual-anti-Exos.

Hence, we designed an exosome-based drug delivery platform that could simultaneously carry multiple immune drugs to perform combination tumor immunotherapy. The endogenic carrier was of low toxicity, low immune rejection, and high stability and enables combination cancer immunotherapy. The combination of anti-PD-L1 and anti-CD40 could greatly improve the effect of immunotherapy compared to direct injection of multiple immune checkpoint drugs. Firstly, two antibodies (anti-PD-L1 and anti-CD40) were modified on the exosome surface which acted not only as targeting molecules but also as block checkpoint inhibitors and costimulatory molecules for the improvement of the therapeutic effect. For modifying the two antibodies on the exosome surface, lipophilic DSPE-PEG-anti-CD40 and DSPE-PEG-PLGVA-anti-PD-L1 were synthesized, respectively, and incubated with donor cells together with immune drugs 2′-3′-cyclic guanosine monophosphate–adenosine monophosphate (cGAMP) (Scheme 1(a)). The antibodies then were modified on the surface of donor cells due to the cell membrane insertion of lipophilic bullet DSPE, meanwhile the drug cGAMP could be uptaken by donor cells. When the cells secrete exosomes, DSPE-PEG-anti-CD40 and DSPE-PEG-PLGVA-anti-PD-L1 on the cell surface together with the drugs can all be transferred to the secreted exosomes. Thus, dual-targeting exosome-loaded drug cGAMP (cGAMP@dual-anti-Exos) was obtained. When the cGAMP@dual-anti-Exos aggregate in tumor tissue, peptides PLGVA connected with anti-PD-L1 will be cut off by the abundant matrix metalloproteinase enzyme (MMP-2) in the tumor microenvironment, so that anti-PD-L1 can separate from the exosomes and bind to the PD-L1 receptor of tumor cells to block the immune checkpoint molecules (Scheme 1(b)). Subsequently, the other antibody on the surface of exosome, anti-CD40, will guide the exosomes to target to CD40 receptors on the DC membrane, and the first positive costimulatory signal will be received by DCs. Then, the exosomes will be uptaken by DCs and release the cGAMP by lysosome-mediated permeabilization of exosomes [27], which can provide the second positive signals to DCs for production of type I interferon (IFN-I) andproinflammatory cytokines [28, 29]. Through the twice activation of dendritic cells (DCs) and the blocking of tumor cell PD-L1, the efficacy of combination cancer immunotherapy for tumor inhibition has been improved. The established cGAMP@dual-anti-Exos utilizes biocompatible exosomes as drug carrier to transmit multiple immune drugs to the target site and simultaneously realize activation of immune response and inhibition of immune escape.

## 2. Results

### 2.1. Preparation and Characterization of cGAMP@dual-anti-Exos

**Figure 1:**
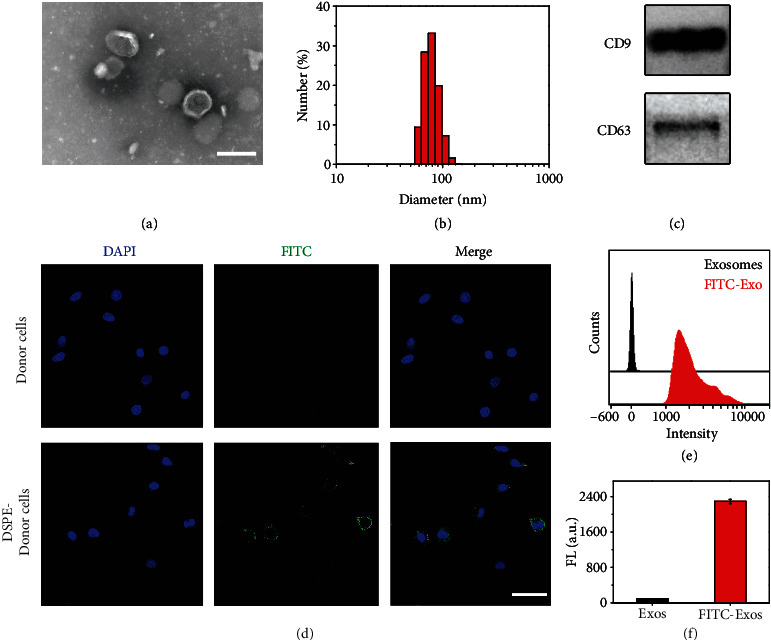
Characterization of exosomes and verification of DSPE insertion. (a) TEM image of extracted exosomes. Scale bar: 200 nm. (b) Particle size distribution of exosomes determined by DLS. (c) Electrophoretogram of CD9 and CD63 in exosomes measured by WB. (d) Confocal fluorescent images of 10 *μ*g/mL DSPE-PEG-bio-treated donor cells and untreated donor cells at 37°C for 2 h. And then, 2 nM SA-FITC was added for the fluorescent label. Scale bar: 50 *μ*m. (e) Flow cytometry analysis of exosomes derived from DSPE-treated donor cells and untreated donor cells. (f) Mean fluorescent intensity histogram quantified from (e).

Firstly, human umbilical vein endothelial cells (HUVECs) were chosen as the donor cells for exosome production, because some studies indicated that exosomes of HUVECs had good performance in tumor therapy [24]. We extracted HUVEC-derived exosomes using supercentrifugation and carried out a series of characterization. The purified exosomes show intact and typical cup-shaped morphology in transmission electron microscopy (TEM) images (Figure 1(a)), and their mean diameter is approximately 80 nm determined by dynamic light scattering (DLS) (Figure 1(b)), which is consistent with the reported range of 40-160 nm [30]. Furthermore, two typical exosome marker proteins CD9 and CD63 were determined by Western blot (WB) (Figure 1(c)). By nanoparticle tracking analysis (NTA) and bicinchoninic acid (BCA) assay, the concentration of extracted exosomes is measured to be 1.533 × 10^9^ particles/mL and the total protein concentration is 0.6808 mg/mL (Figure S1). Through the above morphological observation, dimensional measurement, and protein characterization of extracted exosomes, it is proved that by this method, intact and purified exosomes can be obtained and used for next modification.

To achieve the targeting of both tumor cells and DCs, exosomes should be simultaneously modified with two antibodies anti-PD-L1 and anti-CD40. Firstly, by use of streptavidin (SA) as a bridge, two biotinylated antibodies anti-CD40-bio and anti-PD-L1-bio can be connected to DSPE-PEG-biotin and DSPE-PEG-PLGVA-biotin several times. Then, we could obtain DSPE-PEG-bio-SA-bio-anti-CD40 (simplified as DSPE-PEG-aCD40) and DSPE-PEG-PLGVA-bio-SA-anti-PD-L1 (simplified as DSPE-PEG-PLGVA-aPD-L1) (Figure S2). Before synthesis and extraction of exosomes modified with both aCD40 and aPD-L1, we firstly verified the feasibility of insertion of plug DSPE into the donor cell membrane, which was the key for the production of cGAMP@dual-anti-Exos. We incubated donor cells with DSPE-PEG-biotin and added 2 nM streptavidin-fluorescein isothiocyanate (SA-FITC) for fluorescent labeling. In confocal fluorescence images (Figure 1(d)), the donor cells produce green fluorescence on the cell membrane, demonstrating that DSPE-PEG-biotin can insert into the cell membrane and be used for subsequent production of modified exosomes. We then continued to gather the exosomes produced by the above DSPE-PEG-biotin-inserted cells for verifying that the secreted exosomes also maintained insertion of DPSE. In Figure S3, several green bright spots can be clearly observed, proving that DSPE-PEG-biotin still links to the membrane of cell-derived exosomes. The results from flow cytometry also demonstrate the same conclusion (Figures 1(e) and 1(f)). The above results prove that DSPE can insert into the donor cell membrane and can be transferred to the secreted exosomes.

For collection of enough DSPE-inserted exosomes, we explored the optimal incubation time of DSPE-PEG-biotin and donor cells. As the increase of the incubation time (0–6 days), fluorescence intensity gradually increases, indicating that the amount of DSPE-PEG-biotin inserted into the cell surface increases (Figure S4). However, during this process, the cell viability decreases significantly (Figure S5). This is not conducive to obtain more exosomes. Considering the balance of cell activity and modification time, we finally selected 4 days as the optimal conditions. At this time, more DSPE-PEG-biotin could insert into the cell membrane and donor cells could stay alive for secreting more exosomes.

**Figure 2:**
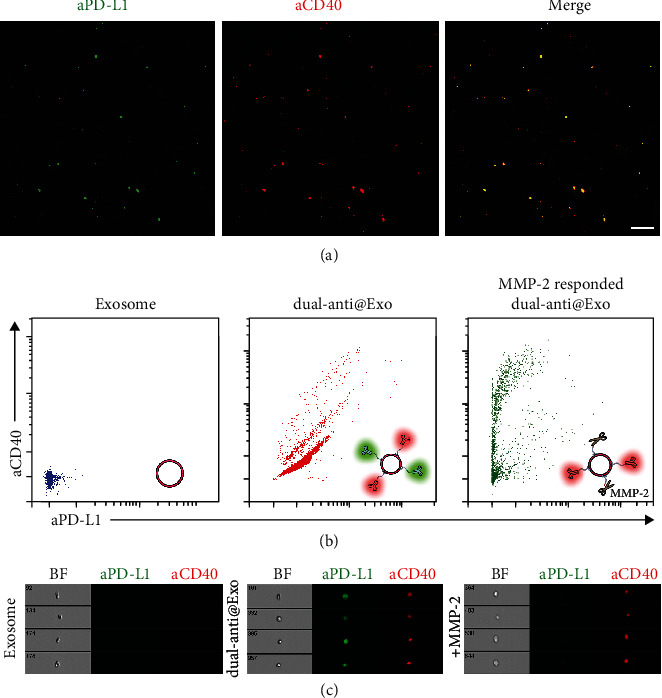
(a) The confocal fluorescent images of dual-anti-Exos. The red fluorescence represents DSPE-PEG-aCD40-Alexa 647 and the green fluorescence represents DSPE-PEG-PLGVA-aPD-L1-Alexa 488. Scale bar: 2 *μ*m. (b) Scatter diagram of exosomes, dual-anti-Exos, and MMP-2-responded dual-anti-Exos detected by flow cytometry. Inset figures: schematic diagram of the three different kinds of exosomes measured by flow cytometry. (c) Flow cytometry images of exosomes, dual-anti-Exos, and MMP-2-responded dual-anti-Exos.

After the above exploration, we try to construct dual-targeting exosomes. DSPE-PEG-aCD40 and DSPE-PEG-PLGVA-aPD-L1 were coincubated with donor cells for the above optimal time of 4 days. After ultracentrifugation, we acquired dual-targeting exosomes (dual-anti-Exos). For verification of the successful production of dual-anti-Exos, we synthesized DSPE-PEG-aCD40-Alexa 647 and DSPE-PEG-PLGVA-aPD-L1-Alexa 488 in the solution and extracted fluorescent-labeled exosomes using the same method. As confirmed by confocal fluorescent imaging (Figure 2(a)), the fluorescence of Alexa 488 and Alexa 647, which come from aPD-L1 and aCD40, respectively, are both observed and are almost completely overlapped in obtained dual-anti@Exos. The same results can also be acquired in the flow cytometry analysis (Figures 2(b) and 2(c)). The two colors can be observed on exosomes and the fluorescence is relatively stronger than that of the control group (Figure 2(b)). Hence, exosomes secreted by the antibody-linked donor cells are successfully modified with the two antibodies on their surface. What is more, after adding 500 ng/mL matrix metalloproteinases 2 (MMP-2), which is well known as overexpressed in the tumor microenvironment [31], only red fluorescence still stayed on dual-anti@Exos while green fluorescence was weakened. This is the result of MMP-2-mediated cleavage of peptide PLGVA. The above results proved that the dual-anti-Exo was successfully constructed and it could respond to MMP-2 in the tumor microenvironment.

In order to enhance immune activity of DCs, we added the drug cGAMP in the process of coincubation between DSPE-antibodies and donor cells. After the modification of antibodies and uptake of cGAMP, donor cells would secrete dual-targeting and drug-loaded exosomes (cGAMP@dual-anti-Exos). For validation of the successful package of cGAMP, the content of cGAMP in exosomes was detected by high-performance liquid chromatography (HPLC). In Figure S6B, the content of drugs in exosomes secreted by different concentrations of cGAMP-treated donor cells was calculated by the standard curve (Figure S6A). Considering the cell viability of donor cells gradually decreasing as the concentration of added cGAMP increased (Figure S7), we finally chose 200 *μ*g/mL as the optimal incubation concentration to obtain more drug-contained exosomes. Hereto, the cGAMP@dual-anti-Exos were successfully constructed.

### 2.2. The Tumor Targeting and Immune Activation Ability of cGAMP@dual-anti-Exos *In Vitro*

**Figure 3:**
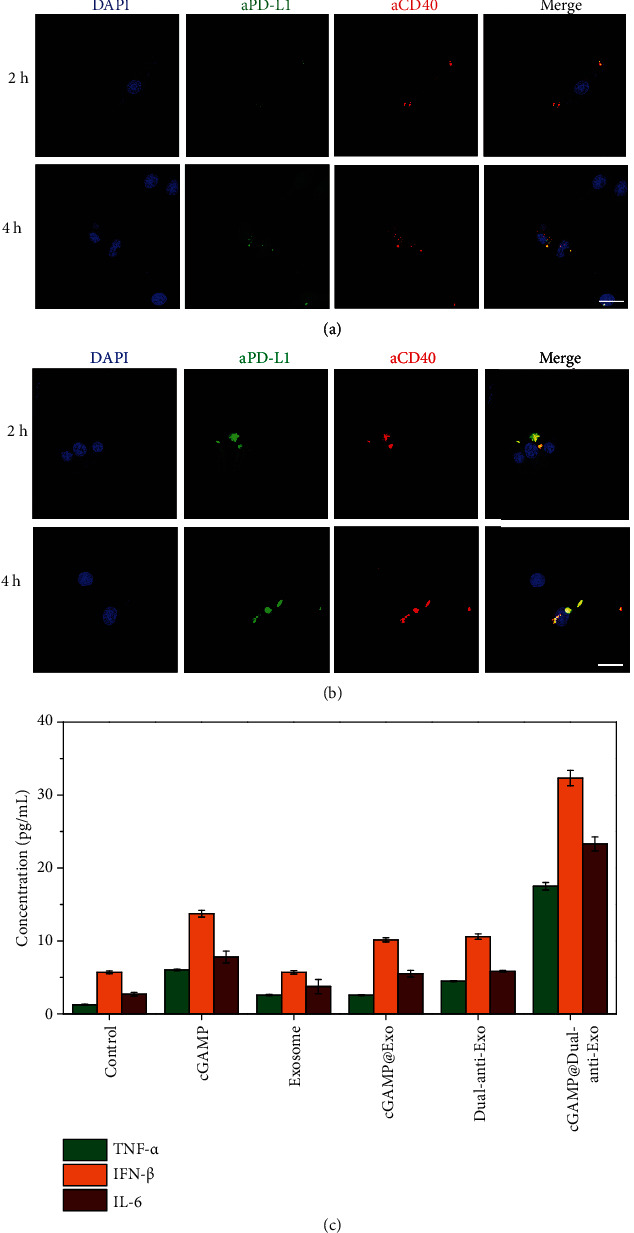
Confocal fluorescent images of B16F10 cells (a) and DCs (b) targeted by cGAMP@dual-anti-Exos. The cells were all incubated with cGAMP@dual-anti-Exos at 37°C for different times. The red fluorescence represents anti-CD40 and green fluorescence represents anti-PD-L1 linked to exosomes separately. The blue fluorescence represents cell nucleus stained by DAPI. Scale bar: 50 *μ*m. (c) The expression level of TNF-*α*, IFN-*β*, and IL-6 under different stimulated conditions at 37°C for 24 h.

The targeting ability of cGAMP@dual-anti-Exos should be assessed. There are two targets of constructed cGAMP@dual-anti-Exos, including immune cell DCs and tumor cell B16F10. After coincubation of cGAMP@dual-anti-Exos and melanoma B16F10 cells for 2 hours, the red fluorescence comes from anti-CD40 and green fluorescence comes from anti-PD-L1 which can be both observed in tumor cells (Figure 3(a)). Furthermore, the red fluorescence and green fluorescence are overlapped and gathered inside the cells and fluorescence improves as the incubation time increases. Similar results can also be noticed in another target DCs (Figure 3(b)). Additionally, it seems that the viability of B16F10 cells are not influenced by the added cGAMP@dual-anti-Exos. It is because of the absence of activated DCs and T cells, which play a major role in tumor elimination. Consequently, the constructed cGAMP@dual-anti-Exos have the ability to target tumor cells as well as immune cell DCs.

Except for targeting, the main task of cGAMP@dual-anti-Exos is the activation of immune cell DCs twice. Thus, we next explored the activation of DCs by evaluating the expression of inflammatory factors (TNF-*α*, IFN-*β*, and IL-6) produced by activated DCs with ELISA (Figure 3(c)). The results show that compared to empty exosomes, the addition of 10 *μ*g cGAMP has an effect on the activation of DCs, which induced slightly the increase of TNF-*α*, IFN-*β*, and IL-6. What is more, dual-targeting empty exosomes (dual-anti-Exos) can also induce DCs to produce a small amount of inflammatory factors, indicating that the combination between anti-CD40 and CD40 can partly promote the activation of DCs. The results reflected that cGAMP and anti-CD40 can mildly activate DCs, whereas the cGAMP@dual-anti-Exos treatment groups produced the highest concentrations of TNF-*α*, IFN-*β*, and IL-6, which is the result of the stimulation of the drug cGAMP and anti-CD40 twice. Thus, the designed dual-anti- and drug-loaded exosomes can induce strong immune response by costimulation of multiple kinds of molecules. Generally, the above confocal fluorescent imaging and cytokine analysis together verify that the constructed cGAMP@dual-anti-Exos can not only target to tumor cells and immune cell DCs but also can promote the activation of DCs and secretion of abundant cytokines, which is very important for subsequent *in vivo* precise cancer immunotherapy.

### 2.3. Tumor Inhibition of cGAMP@dual-anti-Exos *In Vivo*

**Figure 4:**
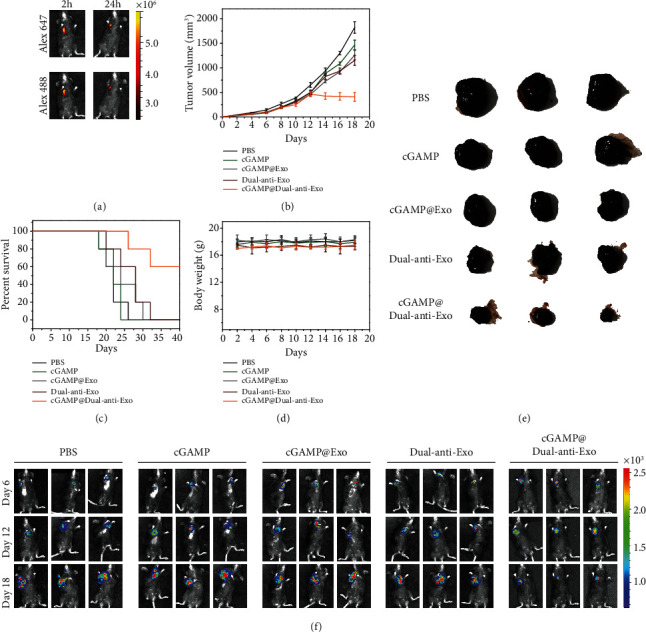
(a) *In vivo* fluorescence images of mice injected with cGAMP@dual-anti-Exos at different times. The fluorescence signal of Alexa 647 was collected under the excitation and emission wavelengths of 620 nm and 670 nm, respectively. The fluorescence signal of Alexa 488 was collected under the excitation and emission wavelengths of 460 nm and 520 nm, respectively. (b) The tumor volume changes of tumor-bearing mice after intratumor injection of different drugs. The data was recorded every two days. (c) The survival rate of different drug-treated tumor-bearing mice. (d) The body weight changes of tumor-bearing mice after intratumor injection of different drugs. (e) The photos of typical tumors dissected from mice with different treatment at day 18. (f) Bioluminescence imaging of tumor-bearing mice after intratumor injection of different drugs.

After demonstrating the great potential of exosomes in tumor targeting and therapeutic drug delivery *in vitro*, we next performed a series of *in vivo* studies for evaluating the therapeutic efficacy by a B16F10 melanoma tumor model. On day 0, C57BL/6 mice were injected with 1 × 10^6^ B16F10-luc tumor cells in the armpit of the right forelimb of mice. Five days after tumor inoculation, cGAMP@dual-anti-Exos were injected into mice and the fluorescent signals of Alexa 488 that came from anti-PD-L1 and Alexa 647 that came from anti-CD40 were measured. The tumor site shows strong fluorescent signal of both the two fluorophore at 2 hours postinjection, and even at 24 hours, the fluorescence still exists and assembles in the tumor site, indicating prolonged blood circulation and good tumor targeting ability of the designed cGAMP@dual-anti-Exos (Figure 4(a)). Tumor growth was monitored by bioluminescence imaging and changes of the tumor volume. Notably, cGAMP@dual-anti-Exos treatment groups show the slowest rate of tumor progression and the smallest tumor volumes (Figures 4(b) and 4(f)). By contrast, the tumor volume of other treatment groups (cGAMP and cGAMP@Exos) increased sharply and the tumors collected at the endpoint of treatment were larger than those of the cGAMP@dual-anti-Exos treatment group (Figure 4(e)). Dual-anti-Exos treatment groups have little effects on tumor, but far less than cGAMP@dual-anti-Exos treatment groups, showing that activation of DCs by cGAMP is a critical factor for the improvement of antitumor immune response. Except for the significant delay of tumor growth, mouse survival is also an extension after cGAMP@dual-anti-Exos treatment (Figure 4(c)). Other treatment groups barely affect mouse survival. 60 percent of the mice are still alive after cGAMP@dual-anti-Exos treatment while the mice that received other drug treatment (Figure 4(c)) are all dead before day 40. Generally, the above results show that the absence of any one of the exosomes (carrier), antibodies (target and drugs), or cGAMP (drugs) will reduce the antitumor effect. In addition, during the treatment, the body weight of all mice was recorded continually and no obvious loss was observed in all groups (Figure 4(d)), suggesting that the immunotherapy based on dual-targeting and drug-loaded exosome was well biocompatible and had great effects on tumor inhibition.

**Figure 5:**
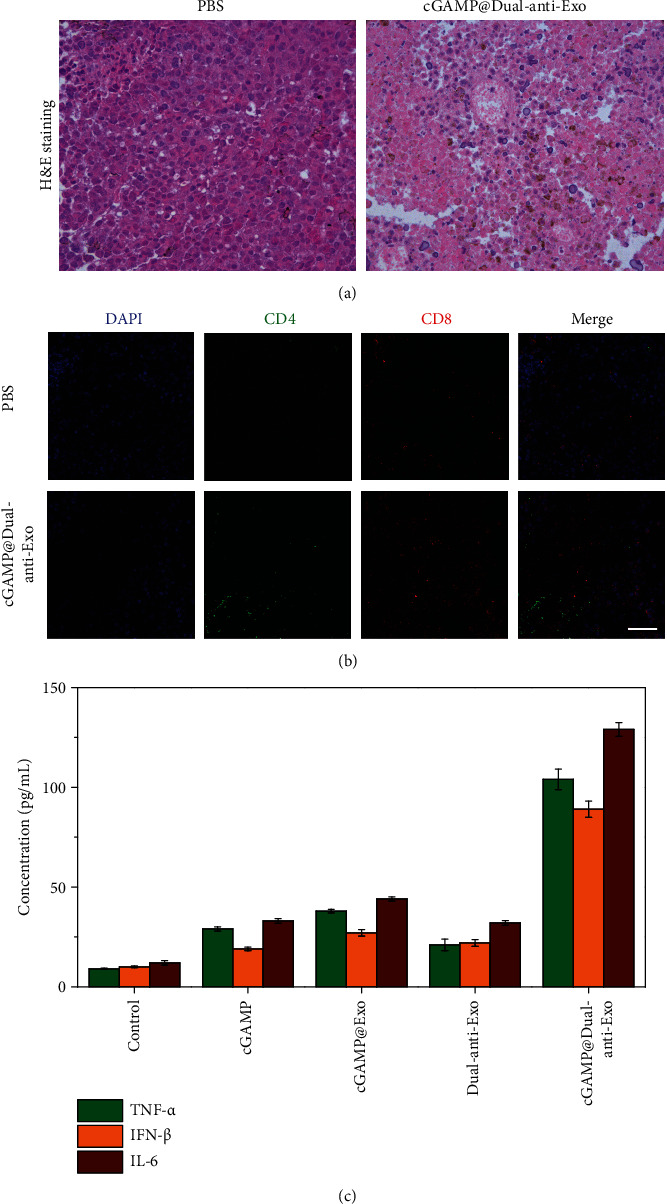
(a) H&E staining of tumor sections from tumor-bearing mice after intratumor injection of different drugs. (b) Immunofluorescence staining of the tumor sections. Red and green show CD8+ T cells and CD4+ cells, respectively, and blue shows the cell nucleus stained with DAPI. Scale bar: 100 *μ*m. (c) The expression level of TNF-*α*, IFN-*β*, and IL-6 in peripheral blood of tumor-bearing mice after intratumor injection of different drugs.

To further investigate the apoptosis of tumor cells and the immunologic mechanisms behind that, we obtained tumor biopsy and carried out H&E staining and immunofluorescence imaging analysis. In Figure 5(a), compared to the control group, the cGAMP@dual-anti-Exos treatment group shows shrinkage of the nucleus and larger area of tumor necrosis, which are consistent with the result of bioluminescence imaging and the tumor size (Figures 4(b), 4(e), and 4(f)). Whether the tumor necrosis was induced by the activated immune system was further revealed by immunofluorescence imaging and the expression of inflammatory factors. CD4 and CD8 receptors were stained in the tumor sections, which had been proved to be crucial molecules of immune system initiation [32]. Compared to the control group, in the cGAMP@dual-anti-Exos treatment group, CD8+ and CD4+ T cells remarkably infiltrate tumor issues (Figure 5(b)). The density of tumor-infiltrated CD8+ T cells was significantly enhanced in cGAMP@dual-anti-Exos treatment groups, while the control groups had limited CD8+ T cell infiltration. Thus, the delivery of cGAMP@dual-anti-Exos increases T-cell activation and expansion, which ultimately suppresses the tumors in mice.

The results of increased expression of inflammatory factors *via* ELISA also confirmed the activation of the immune system. We chose serum TNF-*α*, IFN-*β*, and IL-6 as the investigated cytokines, which were secreted by the activated immune cells and essential factors to eliminate tumor cells [33, 34]. The concentrations of the three cytokines in the cGAMP@dual-anti-Exos-treated group are much higher than that of other control groups (Figure 5(c)). In the individual cGAMP drug group and dual-anti-Exos treatment groups, the concentrations of TNF-*α*, IFN-*β*, and IL-6 are slightly higher than those of PBS control groups but are still far less than those of the cGAMP@dual-anti-Exos-treated groups. Furthermore, the three inflammatory factors in other groups are all relatively low. In general, this developed dual-targeting and drug-loaded exosomes that possess the capacity to deliver drugs to the tumor and eliminate tumor cells by triggering strong antitumor immune response and suppressing tumor immune escape.

## 3. Discussion

In conclusion, based on bio-endogenic exosomes, we constructed a functionalized drug carrier that can simultaneously carry multiple immune drugs to perform combination tumor immunotherapy. The developed drug carrier can specifically target DCs and tumor cells by modified anti-CD40 and anti-PD-L1. Meanwhile, immune response can be greatly enhanced by the loaded immune drugs cGAMP and anti-CD40. Furthermore, tumor immune escape can be prevented by anti-PD-L1, which is deviating from the functionalized exosomes by the cleavage of MMP-2-responded polypeptide. Thus, by the combination of the activation of the immune response and inhibition of immune escape, the tumors are greatly inhibited in virtue of the endogenic low-toxicity exosomes. The developed strategy can also be expanded to other areas for low-toxicity and high-efficiency drug delivery.

## 4. Materials and Methods

### 4.1. Materials and Reagents

Biotin-functionalized phosphatidylethanolamine 1,2-distearoyl-sn-glycero-3-phosphoethanolamine-N-[biotinyl (polyethylene glycol) 2000] (DSPE-PEG-biotin) and DSPE-PEG-PLGVA-biotin were purchased from Xi'an ruixi Biological Technology Co. Ltd. (Xi'an, China). Mouse PD-L1 and CD40 antibodies (aPD-L1, aCD40) were purchased from BioXCell (New Hampshire, USA). Biotin-aPD-L1, biotin-aCD40, biotin-aPD-L1-Alexa-fluor 488, and biotin-aCD40-Alexa-fluor 647 were synthesized by Sangon Biotech Co. Ltd. (Shanghai, China). Streptavidin-coupled FITC (SA-FITC) and SA were purchased from Sangon Biotech Co. Ltd. (Shanghai, China). 2′3′-cGAMP was obtained from InvivoGen (San Diego, CA, USA). CD9 and CD63 antibodies for Western blot (WB) were purchased from Santa Cruz Biotechnology (Dallas, TX, USA). All other reagents used for WB were purchased from Beyotime Biotechnology (Jiangsu, China). IL-6 and TNF-*α* ELISA kits were purchased from Novus Biologicals (Colorado, USA), and IFN-*β* ELISA kit was purchased from InvivoGen (San Diego, CA, USA). All cell culture media and buffers were purchased from HyClone (Logan, Utah, USA), including penicillin mixture (100x), phosphate physiological buffer (1x), fetal bovine serum, DMEM medium, 1640 medium, and trypsin. Enhanced cell counting kit-8 (CCK-8) was purchased from Shanghai Saint-Bio Biotechnology Co. Ltd. (Shanghai, China). Ammonium molybdate-negative staining solution was bought from Shanghai Solarbio Bioscience & Technology Co. Ltd. (Shanghai, China). L-Luciferin was bought from Beyotime Biotechnology (Jiangsu, China).The water used in the experiment is Wahaha purified water (Wahaha, Hangzhou, China). All reagents are of analytical purity.

### 4.2. Cell Culture

The mouse melanoma cell line B16F10-luc was purchased from Shanghai Fuheng Biological Technology (Shanghai, China). B16F10-luc cells were cultured in RPMI 1640 medium containing 10% fetal bovine serum (FBS) and 1% penicillin mixture. The mouse bone marrow-derived dendritic cell line (DC2.4) was purchased from Guangzhou Peiyu Biotechnology Co. Ltd. (Guangzhou, China). DC2.4 cells were kept in DMEM medium supplemented with 10% FBS and 1% penicillin mixture. HUVECs were incubated in endothelial cell medium with 10% FBS, 1% endothelial cell growth factor, and penicillin mixture. All of the cells were incubated in a constant temperature cell incubator (MCO-15AC, SANYO) at 37°C, 5% CO_2_, and 95% air and passaged every 2–3 days.

### 4.3. Extraction of Exosomes

The exosomes were extracted and purified by multistep density gradient ultracentrifugation. After donor cells adhered for 24 h, the cell culture medium was collected and centrifuged to remove cell debris (2000 g for 15 min).Then, the supernatants were filtered with a 0.22 *μ*m filter and the filtrate was collected in an ultracentrifuge tube and centrifuged at 100000 g for 60 min to pellet exosomes using a XPN-100 Ultracentrifuge (Beckman Coulter, USA). Subsequently, the supernatants were removed and exosome pellets were washed with PBS buffer and centrifuged again at 100000 g for 60 min to obtain exosomes with higher purity. The obtained exosomes were resuspended in PBS buffer and stored at −80°C or used freshly.

### 4.4. Characterization of Exosomes

For TEM analysis, a drop of the exosome suspension sample was firstly deposited on a carbon film mesh copper grid and stained by 3% ammonium molybdate negative staining solution. After the sample was dried, the morphology of the exosomes would be observed by HT7700 Transmission Electron Microscope (Hitachi High-Tech, Japan). The hydrodynamic diameter of exosomes was measured by a Malvern Zetasizer Nano (Malvern Instruments, UK).

Western blot (WB) was used for the identification of proteins on exosomes. Firstly, RIPA lysate was added to 50 *μ*L isolated exosome suspension to lysate them. Prior to protein separation, each protein sample was mixed with protein loading buffer and denatured by boiling at 95°C for 5 min. Equal amounts of proteins were loaded into 12.5% SDS-polyacrylamide gels for electrophoretic separation and then transferred onto PVDF membranes. The immunoblots were blocked with blocking buffer and incubated with the primary antibodies at 4°C overnight. The PVDF membranes were washed three times in washing solution, and then, secondary antibodies were added to incubate for 1 h. Finally, the ECL chemiluminescence reagent was added onto the membrane and the visible chemiluminescence signal was collected by the ChemiDoc™ Touch Imaging System (Bio-Rad, Hercules, CA, USA).

### 4.5. Preparation of DSPE-PEG-Biotin-Inserted Donor Cells and Exosomes

The adherent-growing HUVECs were incubated in complete growth medium containing 10 *μ*g/mL DSPE-PEG-biotin. After a period of time, the upper culture medium was collected to obtain DSPE-PEG-biotin-inserted exosomes by the above centrifugation process and adherent cells were washed with PBS buffer three times. Subsequently, 2 nM SA-FITC was added into adherent cells and extracted DSPE-PEG-biotin-inserted exosomes, respectively. After 30 minutes at 37°C, the DSPE-PEG-biotin-inserted cells and exosomes were both washed with PBS buffer 3 times and would be used for subsequent flow cytometry or confocal fluorescent imaging analysis.

### 4.6. Preparation of cGAMP@dual-anti-Exos

We first incubated DSPE-PEG-PLGVA-biotin or DSPE-PEG-biotin (10 *μ*g/mL) with SA for 30 minutes and then added the biotin-PD-L1-Alexa 488 or biotin-CD40-Alexa 647 to incubate for another 30 minutes, and we could obtain DSPE-PEG-PLGVA-biotin-SA-biotin-PD-L1-Alexa 488 (abbreviated to DSPE-PEG-PLGVA-PD-L1-Alexa 488) and DSPE-PEG-biotin-SA-biotin-CD40-Alexa 647 (abbreviated to DSPE-PEG-CD40-Alexa 647) several times. Next, the adherent growing HUVECs were incubated in complete growth medium containing DSPE-PEG-CD40-Alexa 647, DSPE-PEG-PLGVA-PD-L1-Alexa 488, and 200 *μ*g/mL cGAMP. After several days, the upper culture medium was collected to extract cGAMP@dual-anti-Exos by the above centrifugation process. Then, the collected cGAMP@dual-anti-Exos would be analyzed by flow cytometry or confocal fluorescence microscopy.

### 4.7. Characterization of cGAMP@dual-anti-Exos

For flow cytometry experiments, the modified cells or exosomes were firstly suspended in PBS and detected by an ImageStreamX Mark II Imaging Cytometer (Merck Millipore, Germany). The microbead (Sigma Aldrich, St. Louis, USA) with a diameter of 1 *μ*m was used for the assessment of the exosome size. The signal of FITC and Alexa fluor 488 are acquired by a 488 nm excitation laser and 533/55 filter configuration, and Alexa fluor 647 is acquired by a 642 nm excitation laser and 702/85 filter configuration. After the signal acquisition, the data analysis was carried out with IDEAS software.

For confocal fluorescent imaging analysis, the stained cells or exosomes were washed with PBS 3 times to remove excess fluorescent dye firstly. Finally, the Leica TCS SP8 confocal laser scanning microscope (Leica Co. Ltd. Germany) was used to obtain fluorescence images of cells or exosomes. The fluorescence intensity of FITC/Alexa Fluor 488 was excited by the excitation light with a wavelength of 488 nm and the collection range was 500–570 nm and the fluorescence intensity of Alexa fluor 647 was excited by 633 nm excitation light and the collection range was 650–710 nm.

### 4.8. Cell Viability Assay

To investigate the cytotoxicity of DSPE-PEG-bio and drug cGAMP, the Cell Counting Kit-8 (CCK-8) assay was performed according to the manufacturer's instructions. Firstly, the donor cells were seeded into a corning 96-well microtiter plates. After incubated with DSPE-PEG-bio for different days, 10 *μ*L CCK-8 was added and incubated with adherent cells at 37°C for an hour. At last, optical difference (OD) which represents cell viability was read at 490 nm by a RT 6000 microplate reader (Rayto, USA). The cytotoxicity of different concentrations of cGAMP was also assessed according to the above procedure.

### 4.9. Evaluation of Cytokines Secreted by DC2.4 Cells

Firstly, we obtained cGAMP@dual-anti-Exos, dual-anti-Exos, cGAMP@Exos, and empty Exos according the above steps. Then, we added these nanodrugs (0.12 nM) and cGAMP (1 *μ*M) into bone marrow-derived dendritic cells (DC2.4). After 24 hours, the supernatant was collected and the expression levels of interferon TNF-*α*, IFN-*β*, and IL-6 were evaluated by a RT 6000 microplate reader according the ELISA kit operation manuals.

In in vivo tumor models, six- to eight-week-old female C57BL/6 mice were purchased from Jinan Pengyue Experimental Animal Breeding Corporation (Jinan, China). All performed mouse studies were approved by the Principles of Laboratory Animal Care (People's Republic of China) and the Guidelines of the Animal Investigation Committee, Biology Institute of Shandong Academy of Science, China. To establish the tumor-bearing mouse model, 1 × 10^6^ B16-F10-luc cells in serum-free RPMI1640 medium were injected subcutaneously into the flanks of the C57BL/6 mice on day 0. Five days after the injection of melanoma cells, PBS, cGAMP (100 *μ*g), cGAMP@exos (200 *μ*g), dual-anti-Exos (200 *μ*g), and cGAMP@dual-anti-Exos (200 *μ*g) were inoculated into mice by intratumor injection on days 8, 11, 14, and 17. The tumor volumes were measured by a vernier caliper and calculated according to the formula volume = (length × width^2^)/2. The body weight of mice was measured every two days for researching the potential toxicities of injected nanodrugs. Mice were euthanized when the tumor volume was larger than 200 mm^2^.

### 4.10. *In Vivo* Imaging Experiment

For imaging tumor in mice, bioluminescence images were acquired with an in vivo imaging system (IVIS Lumina III, US). The mice were intraperitoneally injected with 15 mg/mL L-luciferin in DPBS, and 10 minutes later, the bioluminescence images were collected by Living Image software.

To study the tumor targeting ability of the designed dual-targeting and drug-loaded exosomes, 200 *μ*g cGAMP@dual-anti-Exos were injected into tumor issues and the fluorescence images were collected by an in vivo imaging system (IVIS Lumina III, US). The fluorescence of Alexa 488 and that of Alexa 647 were separately measured at 2 hours and 24 hours after the drug injection.

### 4.11. Hematoxylin and Eosin (H&E) Staining and Immunofluorescence Imaging

The tumors were separated from mice and fixed in 4% formaldehyde. Then, the tumors were dehydrated and embedded in paraffin block. Several micrometer sections were dissected using a cryotome and incubated with H&E or primary antibodies and fluorescence-labeled secondary antibodies. The cell nuclei were stained with DAPI. Finally, slides were analyzed under a Leica TCS SP8 confocal laser scanning microscope.

### 4.12. Cytokine Detection of Serum

The serum samples were collected from mice with different treatments on day 18. The expression levels of interferon TNF-*α*, IFN-*β*, and IL-6 were evaluated by the RT 6000 microplate reader according to the ELISA kit operation manuals.

## Data Availability

All data are available in the manuscript, in supplementary materials, or from the authors.

## Supplementary Materials

Supplementary Materials Figure S1: the protein standard curve was acquired by a BCA Protein Assay Kit. Figure S2: schematic diagram of the synthetic process of DSPE-PEG-aCD40 and DSPE-PEG-PLGVA-aPD-L1. Figure S3: fluorescent images of DSPE-PEG-bio-inserted exosomes produced by donor cells. Figure S4: flow cytometry analysis and mean fluorescent intensity histogram of cells incubated with DSPE-PEG-bio for different times. Figure S5: cell viability of donor cells incubated with DSPE-PEG-bio for different times detected by CCK-8. Figure S6: the standard curve of cGAMP detected by HPLC. (b) The content of cGAMP in exosomes secreted by donor cells incubated with different concentrations of cGAMP. Figure S7: the donor cell viability with different concentrations of cGAMP added detected by CCK-8.
