# Supplementary material for: Responsive Dual-Targeting Exosome as a Drug Carrier for Combination Cancer Immunotherapy
Source: Research (Wash D C). 2021 Aug 31;2021:9862876. doi: 10.34133/2021/9862876 (PMC8426567; doi:10.34133/2021/9862876)
Supplement: Supplementary Materials — Figure S1: the protein standard curve was acquired by a BCA Protein Assay Kit. Figure S2: schematic diagram of the synthetic process of DSPE-PEG-aCD40 and DSPE-PEG-PLGVA-aPD-L1. Figure S3: fluorescent images of DSPE-PEG-bio-inserted exosomes produced by donor cells. Figure S4: flow cytometry analysis and mean fluorescent intensity histogram of cells incubated with DSPE-PEG-bio for different times. Figure S5: cell viability of donor cells incubated with DSPE-PEG-bio for different times detected by CCK-8. Figure S6: the standard curve of cGAMP detected by HPLC. (b) The content of cGAMP in exosomes secreted by donor cells incubated with different concentrations of cGAMP. Figure S7: the donor cell viability with different concentrations of cGAMP added detected by CCK-8. [file 9862876.f1.docx]

Supporting Information for

**Responsive Dual-targeting Exosome as Drug Carrier for Combination Cancer Immunotherapy**

Yuanyuan Fan, Yingshun Zhou, Meng Lu, Haibin Si, Lu Li,* Bo Tang*

Author Email: [tangb@sdnu.edu.cn](mailto:tangb@sdnu.edu.cn), lilu5252@163.com

**Table of Contents**

[**Supplemental Figures**](#_Toc57367406) 6

[Fig. S1. Protein standard curve was aquired by a BCA Protein Assay Kit](#_Toc57367396) 6

[Fig. S2. Schematic diagram of synthetic process of DSPE-PEG-aCD40 and DSPE-PEG-PLGVA-aPD-L1..](#_Toc57367396) 6

[Fig. S3. Fluorescent images of DSPE-PEG-bio inserted exosomes produced by donor cells](#_Toc57367396) 6

[Fig. S4. Flow cytometry analysis and Mean fluorescent intensity histogram of cells incubated with DSPE-PEG-bio for different time](#_Toc57367396) 7

Fig. S5. [Cell viability of donor cells incubated with DSPE-PEG-bio for different time detected by CCK-8.](#_Toc57367396) 7

[Fig. S6. The standard curve of cGAMP detected by HPLC. (B) The content of cGAMP in exosomes secreted by donor cells incubated with different concentration of cGAMP.](#_Toc57367396) 7

[Fig. S7. The donor cell viability with different concentration of cGAMP added detected by CCK-8…](#_Toc57367396) 8

**Supplemental Figures**





**Figure S1.** Protein standard curve was acquired by a BCA Protein Assay Kit. The absorbance was measured at the wavelength of 562 nm by microplate reader and the total protein concentration of exosomes was calculated according to the standard curve.


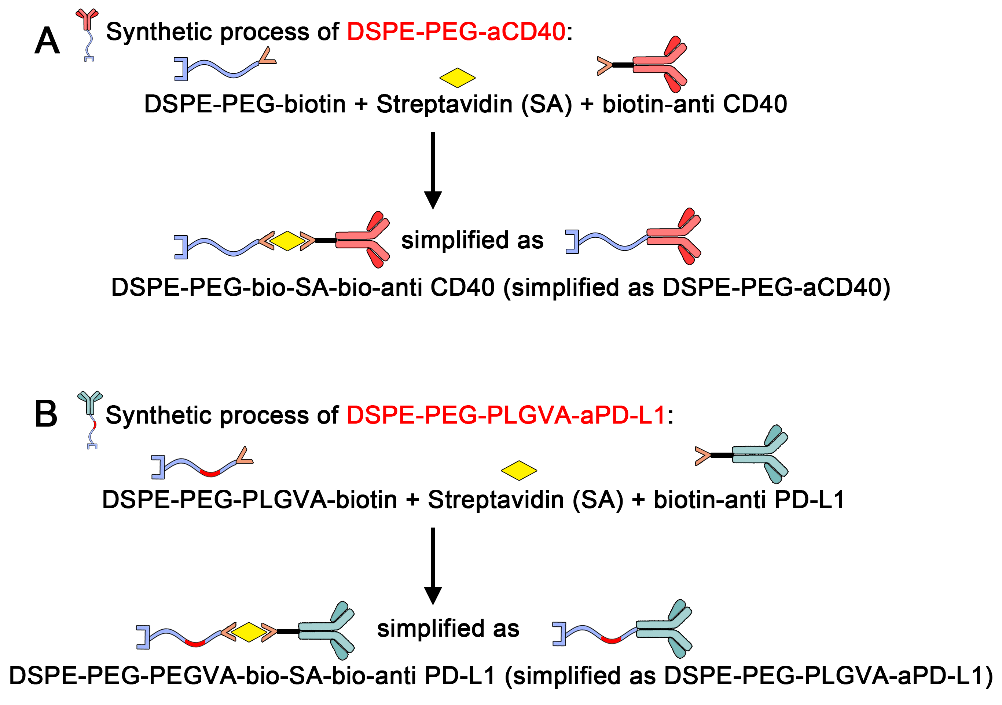


**Figure S2.** Schematic diagram of synthetic process of DSPE-PEG-aCD40 (A) and DSPE-PEG-PLGVA-aPD-L1 (B).


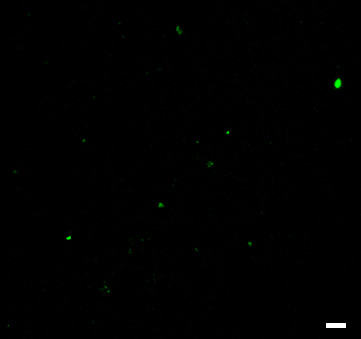


**Figure S3.** Fluorescent images of DSPE-PEG-bio inserted exosomes produced by donor cells. Scale Bar: 400 nm.


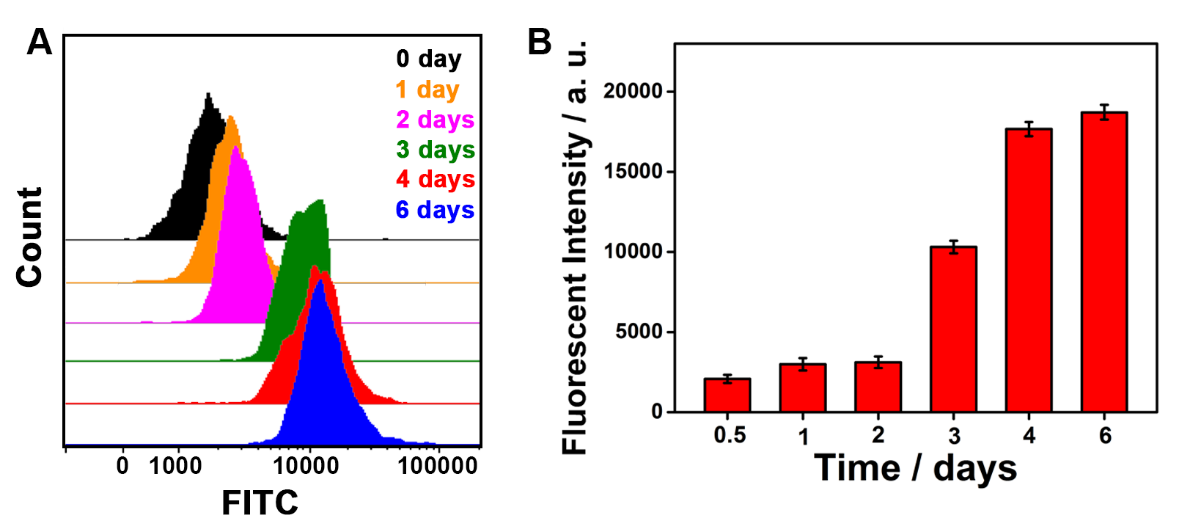


**Figure S4**. Flow cytometry analysis (A) and mean fluorescent intensity histogram (B) of cells incubated with DSPE-PEG-bio for different time.





**Figure S5**. Cell viability of donor cells incubated with DSPE-PEG-bio for different time detected by CCK-8.


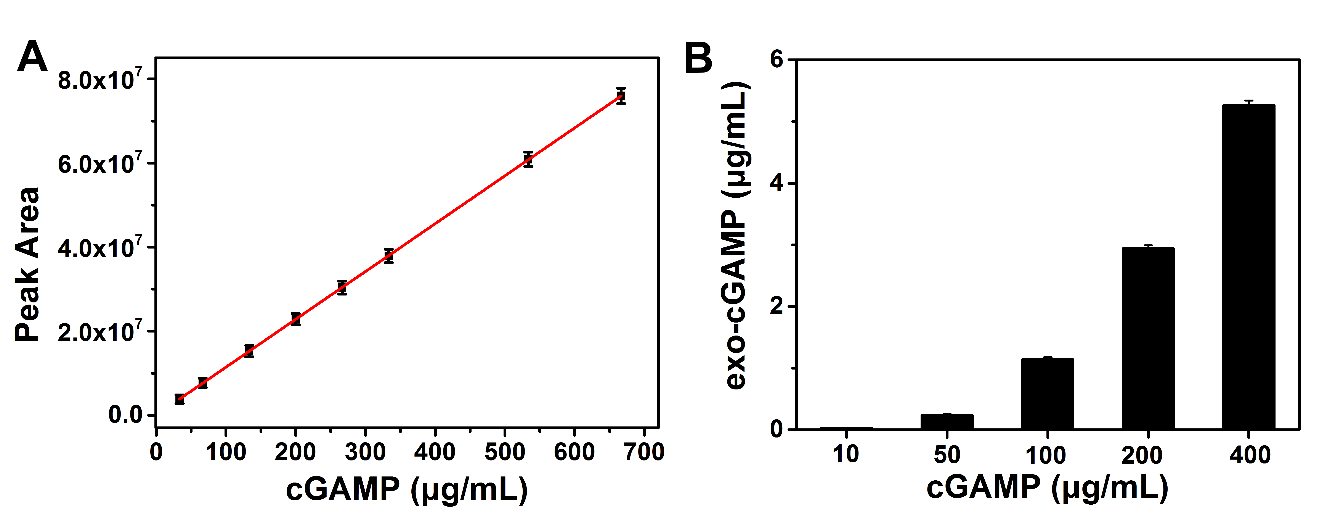


**Figure S6.** (A)The standard curve of cGAMP detected by HPLC. (B) The content of cGAMP in exosomes secreted by donor cells incubated with different concentration of cGAMP.

**

**

**Figure S7.** The donor cell viability with different concentration of cGAMP added detected by CCK-8.
